# 2141. Investigating a (Natural) Killer by Studying the Victims: Searching for Mechanisms to Selectively Inhibit NK-cell Immunosuppressive Function to Improve Vaccine Responses

**DOI:** 10.1093/ofid/ofac492.1761

**Published:** 2022-12-15

**Authors:** Andrew Cox, Stephen N Waggoner, Alexander Katko, Krishna Roskin

**Affiliations:** Cincinnati Children's Hospital Medical Center, Cincinnati, Ohio; Cincinnati Children's Hospital Medical Center/ University of Cincinnati College of Medicine, Cincinnati, Ohio; Cincinnati Children's Hospital Medical Center, Cincinnati, Ohio; Cincinnati Children's Hospital Medical Center, Cincinnati, Ohio

## Abstract

**Background:**

Decades of effort have yet to deliver an efficacious vaccine for pathogens such as HIV, despite major revelations about HIV antigens and development of new vaccine platforms. Innovative outside-the-box approaches are needed to overcome immunological roadblocks to the success of past and current vaccine regimens. We discovered that natural killer (NK) cells are a potent obstacle to vaccine success through perforin-dependent suppression of activated CD4 T cell responses early after vaccination. This killing reduces the quantity and quality of antibody responses. Yet, the features of NK cells that enable this activity or determine susceptibility of specific subsets of T cells remain ill-defined.

**Methods:**

We used transgenic virus-specific mouse CD4 T cells to detect very early changes in responding T cells immediately proximal to immunoregulatory killing by NK cells. Transgenic T cells were seeded in recipient mice, depleted or not of NK cells, and then infected with lymphocytic choriomeningitis virus. At early time points post-infection, these T cells were isolated for single-cell RNA-sequencing or high-dimensional flow cytometry analyses. These data revealed transcriptomic and proteomic features of subsets of activated CD4 T cells whose presence was enabled or enhanced by the absence of NK cells.

**Results:**

Perforin-dependent killing of CD4 T cells by NK cells is measurable on days 2 and 3 post-infection. Analysis of the pool of responding T cells on day 3 revealed phenotypic skewing away from follicular helper T cell-type responses to Th1-type responses in the presence of NK cells. Single cell RNA sequencing and high-dimensional spectral flow cytometry reveal clusters of T cells that are markedly enriched in the absence of NK cells.

**Conclusion:**

We created a model system permitting characterization of the population of T cells targeted by NK cells after infection or immunization. Elucidation of features of the targeted T cells will provide mechanistic insights in the receptor-ligand system enabling this biology, improve our understanding of how NK cells influence the pool of responding T cells, and facilitate discovery of the corresponding subset of NK cells responsible for this activity. These discoveries are likely to facilitate development of methods to enhance vaccine responses.

**Disclosures:**

**All Authors**: No reported disclosures.

